# Waterpipe use among people who smoke in rural Vietnam: Secondary analysis from the M2Q2 randomized clinical trial

**DOI:** 10.18332/tpc/217702

**Published:** 2026-07-16

**Authors:** Andrew W. Liu, Yvonne P. Nguyen, Narayani Ballambat, Hai Tran, Xuan Nguyen, Julie Flahive, Jeroan J. Allison, Nguyen Vu, Hoa L. Nguyen, Rajani S. Sadasivam

**Affiliations:** 1 University of Massachusetts Chan Medical School, Worcester, United States; 2 Department of Population and Quantitative Health Sciences, University of Massachusetts Chan Medical School, Worcester, United States; 3 Institute of Population, Health and Development (PHAD), Hanoi, Vietnam

**Keywords:** cessation, mHealth, Vietnam, rural, waterpipe

## Abstract

**INTRODUCTION:**

Waterpipe (WTP) tobacco smoking is highly prevalent in Vietnam. WTP use is associated with higher nicotine exposure and increased risk of pulmonary and cardiovascular diseases. The aim of this study is to understand WTP use and how it may influence smoking behaviors and cessation.

**METHODS:**

We conducted a secondary analysis of data from the mHealth Messaging to Motivate Quitline Use and Quitting (M2Q2) randomized trial, which evaluated a mobile health peer-texting intervention in Northern Vietnam. Logistic and mixed-effects logistic regression was used to assess the association between WTP, CO-verified cessation, and Quitline use, after accounting for commune-level clustering and adjusting for demographic factors and randomization.

**RESULTS:**

Among 749 male smokers (mean age: 42.7 years), 53% reported WTP use. Higher education (high school: adjusted odds ratio, AOR=0.61; CI: 0.43–0.86, college: AOR=0.47; CI: 0.30 – 0.73), and age >60 years (AOR=0.53; CI: 0.28–0.99) were inversely associated with WTP use, compared to smokers who only completed secondary school, and were aged <30 years, respectively. Compared to non-WTP users, WTP users smoked fewer cigarettes daily (p<0.001), smoked sooner after waking (p<0.001), spent more money weekly on tobacco products (p<0.001), and began smoking at an earlier age (p=0.02). WTP was not significantly associated with CO-verified cessation, and users had lower non-significant Quitline use.

**CONCLUSIONS:**

WTP users differed from non-WTP users in demographic and smoking behaviors. WTP did not significantly affect CO-verified cessation, with a non-significant trend of reduced Quitline use, suggesting barriers to treatment uptake. Tailored cessation strategies that address WTP-specific challenges are needed to improve outcomes in this high-risk group.

## INTRODUCTION

Tobacco use remains a major public health challenge globally, with low- and middle-income countries (LMICs) bearing a disproportionate share of the burden^1^. In countries like Vietnam, tobacco-related prevalence, morbidity, and mortality are particularly high, with an estimated 42.3% of men smoking and approximately 40000 tobacco-related deaths annually^2,3^. Cigarette smoking has been the primary focus of tobacco control interventions, despite the high prevalence of waterpipe smoking (WTP, locally known as *thuốc lào*), a traditional form of tobacco consumption involving inhalation of smoke filtered through water. National data from 2001–2002 estimated a prevalence of 51.2% for WTP use, with higher prevalence in rural versus urban areas^4-7^.

WTP poses significant health risks, with established associations with pulmonary and cardiovascular diseases and cancers^6,8-10^. In a dose–response meta-analysis, WTP significantly increased the overall risk of death by 82% and the risk of dying from all types of cancer by 91% among waterpipe smokers with more than 20 years of smoking history^11^. Additionally, dual use of waterpipes and cigarettes may increase nicotine dependence and compound health risks^9,11-13^.

Despite the established health risks associated with WTP, few studies have examined who uses WTP or how WTP may influence smoking behaviors and cessation efforts. This is a critical gap, as WTP may influence both the intensity of nicotine exposure and the success of cessation interventions. Prior studies suggest that motivation to quit one form of tobacco can positively influence cessation of another^12,13^, underscoring the potential value of tailoring interventions to target WTP. Therefore, understanding the sociodemographic and behavioral dynamics that shape WTP can inform more comprehensive and effective tobacco control strategies in intervention settings in LMICs like Vietnam.

In the present study, we conducted a secondary analysis using data from the parent study – mHealth Messaging to Motivate Quitline Use and Quitting (M2Q2) trial in rural Northern Vietnam (2017–2022) – which assessed the effectiveness of a mobile health peer-texting intervention encouraging Quitline use and smoking cessation^14^. In the present study, we explore three research questions: 1) Which sociodemographic factors are associated with WTP use; 2) How smoking behaviors differ by WTP use; and 3) Whether waterpipe use impacts trial outcomes. Addressing these questions will help clarify whether waterpipe users differ in meaningful ways from cigarette-only smokers and whether they may require tailored cessation strategies.

## METHODS

### Study design

We conducted a secondary data analysis using data collected in the parent trial M2Q2, a two-arm double-blinded randomized control trial with smokers recruited at four rural community centers in Hung Yen province in Northern Vietnam (NCT03567993) by the Vietnam Institute of Population, Health and Development (PHAD)^14,15^. Participants and research staff were blinded to allocation to intervention or comparison group via our text messaging system, with research staff also being blinded to allocation while assessing outcomes.

### Ethics

This study involving humans was approved by the UMass Chan and Population, Health, and Development Institution Review Boards and monitored by a Data and Safety Monitoring Board. The studies were conducted in line with local legislation and institutional requirements. Participants provided written informed consent to participate in this study.

### Parent trial

The objective of the parent trial was to examine the effectiveness of a 6-month texting intervention to promote the use of a Vietnam Ministry of Health-based Quitline (Bach Mai Quitline, located in Northern Vietnam) and improve smoking cessation. The intervention group received peer-written motivational text messages promoting Quitline use and smoking cessation for six months, along with biweekly messages assessing smoking status and interest in Quitline referral, while the comparison group received only biweekly smoking status assessments via text messages. The parent trial was conducted among 750 participants, with 1 participant withdrawing at baseline, with 369 participants remaining after six-month follow-up for the intervention group, and 372 participants remaining for the control group. Retention rate was 98.8%. The intervention group had significantly higher verified Quitline use (18% vs 1%) but no significant difference in carbon monoxide–verified smoking cessation rates at six months (both ~28%). The methods and full results of the parent trial have been published^14,15^.

### Setting and population

Participants were recruited at their community health centers by study staff and local collaborators. After providing written consent forms and meeting the eligibility criteria of the study, all participants were administered a baseline survey by trained staff. All survey instruments were translated into Vietnamese by a certified translator. Translators are native Vietnamese speakers and members of the American Translators Association. Translated documents underwent editing by an accredited language expert to confirm accuracy. All translated materials received certification of accuracy^15^. Participant inclusion criteria were: 1) a resident of the selected commune; 2) current smoker; 3) able to receive texts and read text (literate); 4) not cognitively impaired; 5) not a participant who helped develop the motivational texts used in the intervention; and 6) not a family member of another participant in the study. Since rates of smoking in Vietnam are very low for women^16^, sex was not an eligibility criterion for the parent study and resulted in a sample that was 100% male.

### Data collection

Baseline data included demographics and smoking-related behaviors. WTP use was assessed via the question: ‘What other tobacco products do you smoke: Waterpipes? Yes/No’. Baseline data also assessed chronic comorbidities (heart disease, lung disease, hypertension, diabetes, presence of ulcers, kidney disease, liver disease, anemia, cancer, depression, arthritis, back pain, other rheumatic disease), quality of life (QOL), and Smoking Self-Efficacy SEQ-12^17^. The primary outcomes of the parent trial were 6-month CO-verified smoking cessation and Quitline usage, with self-efficacy on the SEQ-12 as a secondary outcome. Smoking cessation was defined using the 7-day point prevalence question at six months, verified by carbon monoxide breath testing (≤6 ppm), and Quitline use was confirmed through call records collected by the Quitline.

### Data analysis

Our analyses corresponded to our research questions. To explore which sociodemographic factors were associated with WTP use, participants’ demographics and smoking characteristics were compared between WTP users and non-WTP users, using chi-squared or Fisher’s exact test for categorical variables and t-test for continuous variables. Baseline variables included age, education level, marital status, number of adults and children in a household, financial anxiety status in the last 12 months, number of chronic diseases, self-perceived health, and inpatient hospitalization in the last six months. Univariate logistic regression was conducted to examine associations between each independent variable and WTP use. Variables were selected for inclusion in the multivariable adjusted logistic regression model determined by both a priori knowledge and statistical criteria (p<0.25 in univariate analysis). Model fit was examined using the goodness-of-fit test and an area under the receiver operator characteristic (ROC) curve. We then examined how smoking characteristics differed between WTP users and non-WTP users. Next, to evaluate the impact of WTP use on trial outcomes, we estimated mixed-effects logistic regression models for: 1) CO-verified smoking cessation at six months, and 2) verified Quitline use. For each outcome, we used both an unadjusted model and an adjusted model. In the adjusted models, WTP status was the primary predictor, and we adjusted for the same covariates identified in the univariate screening (p<0.25), which included age, education level, employment, and comorbidities. All mixed-effects models accounted for clustering at the commune level. Finally, we examined the association between WTP and the Quitline usage group using a similar modeling approach. This analysis was restricted to the intervention group, as only intervention participants received active referral to the Quitline. Baseline analyses were completed with our total sample of 749 participants, while analyses based on follow-up had fewer participants due to loss to follow-up or missing data values.

An alpha level of 0.05 was used as a criterion to determine statistical significance. All analyses were performed using SAS software (version 9.4; SAS Institute, Cary, NC). Complete case analysis was used to manage missing data.

### Additional covariates

We included the following covariates in our model for adjustment: age, education level, employment, and number of chronic diseases. Age was grouped into four categories: 30–39, 40–49, 50–59, and ≥60 years. Education level was also grouped into four categories: Primary school, completed secondary school, completed high school, and vocational college/university or higher. Employment was defined as self-employed, farmer, other, and paid work. Finally, the number of chronic diseases was grouped as: 0, 1, or ≥2 comorbidities. These covariates were selected through both statistical approach and a priori knowledge. Variables such as age, education level, employment, and number of chronic diseases can represent aspects of life outside of the healthcare system that may influence health behaviors and outcomes^18,19^.

## RESULTS

### Sociodemographic factors are associated with WTP use

Out of 749 individuals, a total of 398 individuals who smoke (53%) reported WTP use at baseline. Education level was significantly associated with WTP use. A larger proportion of WTP users completed secondary school compared to non-WTP users (52% vs 38%) (Table 1). Non-WTP users were more likely to have attained higher education levels, such as vocational college or university (20% vs 14%). There was no significant difference in other demographic characteristics between the two groups in terms of age, marital status, employment status, household composition, or health status.

**Table 1:** Demographic and socio-economic characteristics by waterpipe use status at baseline, M2Q2 randomized controlled trial, Vietnam, November 2018–April 2021 (N=749)

*Characteristics*	*Total n*	*Waterpipe use*		*p*
		*Yes (N=398) n (%)*	*No (N=351) n (%)*	
**Age** (years)				0.22
<30	121	67 (17)	54 (15)	
30–39	210	111 (28)	99 (28)	
40–49	182	103 (26)	79 (23)	
50–59	158	85 (21)	73 (21)	
≥60	78	32 (8.0)	46 (13)	
**Education level**				0.003***
Primary school	55	29 (7.3)	26 (7.4)	
Completed secondary school	340	205 (52)	135 (38)	
Completed high school	228	55 (14)	71 (20)	
Vocation college/University or higher	126	109 (27)	119 (34)	
**Marital status**				0.57
Divorced/widowed/separated/single	101	51 (13)	50 (14)	
**Employment**				0.15
Self-employed	177	106 (27)	71 (20)	
Farmer	174	93 (23)	81 (23)	
Other	78	163 (41)	157 (45)	
Paid work	320	36 (9.1)	42 (12)	
**Household members**				
Number of adults, mean (SD)		3.3 (1.4)	3.3 (1.2)	0.89
Number of children, mean (SD)		1.4 (1.3)	1.4 (1.2)	0.95
**Financial anxiety status in last 12 months**				0.90
Never/rarely	406	213 (54)	193 (55)	
Sometimes	150	80 (20)	70 (20)	
Usually/always	193	105 (26)	88 (25)	
Number of chronic diseases*				0.20
0	349	175 (44)	174 (50)	
1	225	130 (33)	95 (27)	
≥2	175	93 (23)	82 (23)	
**Self-perceived health**				0.30
Excellent/very good	161	93 (23)	68 (19)	
Good	547	286 (72)	261 (74)	
Fair	37	16 (4)	21 (6)	
Poor	4	3 (0.75)	1 (0.28)	
**Inpatient hospitalization in the last 6 months**	39	22 (5.5)	17 (4.8)	0.67

* Comorbidities include: heart disease, lung disease, hypertension, diabetes, presence of ulcers, kidney disease, liver disease, anemia, cancer, depression, arthritis, back pain, other rheumatic disease, and other reported problems deemed relevant.

*** p<0.05.

In the models, the older age groups had lower odds of WTP use compared to those <30 years (Table 2). Completing high school (AOR=0.61; 95% CI: 0.43–0.86), or vocational college/university or higher (AOR=0.47; 95% CI: 0.30–0.73) was associated with statistically significant odds of WTP use compared to completing secondary school, while completing primary school was associated with decreased odds, but lacked significance. Individuals reporting at least one comorbidity had higher odds of WTP use compared to those with none, but this was not significant.

**Table 2:** Factors associated with waterpipe usage, M2Q2 randomized controlled trial, Vietnam, November 2018–April 2021 (N=749)

Variables	AOR	95% CI
**Age** (years)		
<30	1.15	(0.72–1.84)
30–39 (ref.)	1	
40–49	0.99	(0.65–1.50)
50–59	0.86	(0.54–1.36)
≥60	**0.53**	(0.28–0.99)*
**Education level**		
Primary school	0.73	(0.41–1.31)
Completed secondary school (ref.)	1	
Completed high school	**0.61**	(0.43–0.86)*
Vocation college/University or higher	**0.47**	(0.30–0.73)*
**Employment status**		
Self-employed	1.08	(0.72–1.62)
Farmer	1.07	(0.60–1.91)
Other	1.42	(0.97–2.07)
Paid work (ref.)	1	
**Number of chronic diseases**		
0 (ref.)	1	
1	**1.43**	(1.00–2.03)*
≥ 2	1.24	(0.84–1.83)

AOR: adjusted odds ratio. Adjusted logistic regression model includes age, education level, employment, and number of chronic diseases. ROC=0.611; Goodness-of-fit test p=0.97.

* p<0.05.

### Smoking behaviors by WTP use

Several tobacco use behavior differences emerged between waterpipe users and non-users (Table 3). WTP users smoked fewer cigarettes per day on average than non-users (11 vs 16) and reported smoking sooner after waking. Specifically, 53% of WTP users smoked within 5 minutes of waking compared to only 22% of non-WTP users (p<0.001). Additionally, WTP users spent significantly more money on tobacco products weekly compared to non-WTP users. WTP users first smoked tobacco at the age of 18 years, while non-WTP users first smoked tobacco at 19 years (p=0.02). Thirty percent of non-WTP users were more likely to work in environments with smoking restrictions compared to 20% of WTP users (p=0.002). Furthermore, baseline smoking self-efficacy scores (SEQ-12) were significantly higher among non-WTP users compared to WTP users (p=0.004).

**Table 3:** Smoking characteristics by waterpipe usage, participants randomized to text-based smoking cessation intervention, M2Q2 randomized controlled trial, Vietnam, November 2018–April 2021 (N=749)

*Characteristics*	*Waterpipe use*		*p*
	*Yes (N=398) n (%)*	*No (N=351) n (%)*	
**Cigarettes per day**, mean (SD)	11 (10)	16 (9.4)	<0.001*
**Age when first smoked tobacco**, mean (SD)	18 (5.7)	19 (6.0)	0.02*
**Number of years of daily tobacco use**, mean (SD)	24 (13)	24 (14)	0.94
**Minutes to first tobacco use**			<0.001*
≤5	212 (53)	76 (22)	
6–30	113 (28)	120 (34)	
31–60	17 (4.3)	29 (8.3)	
>60	56 (14)	126 (36)	
**Smoke other tobacco products** (non-waterpipe)	398 (100)	2 (0.57)	<0.001*
**Tried an e-cigarette, even just once**	80 (20)	60 (17)	0.29
**Workplace has rules about smoking tobacco**	79 (20)	104 (30)	0.002*
**Money per week currently spend on tobacco products** (VND)			<0.001*
<10000	100 (25)	13 (3.7)	
10000–20000	30 (7.5)	18 (5.1)	
20000–30000	47 (12)	39 (11)	
30000–40000	37 (9.3)	54 (15)	
>40000	184 (46)	227 (65)	
**Quit attempt in past 12 months**	223 (56)	192 (55)	0.71
**Before being contacted for this survey, I had heard of the Bach Mai Quitline**	63 (16)	64 (18)	0.38
**Besides myself, there is at least one person who lives in my home that currently smokes tobacco**	125 (31)	95 (27)	0.19
**Baseline Smoking Self-Efficacy SEQ-12**, mean (SD)	32 (9.3)	34 (10)	0.004*

VND: 1000 Vietnamese Dongs about US$0.038.

* p<0.05.

### WTP use and smoking cessation

WTP users had lower odds of CO-verified smoking cessation than non-users (Table 4).

**Table 4:** Association between waterpipe usage and smoking cessation among all participants using mixed-effect modeling, M2Q2 randomized controlled trial, Vietnam, November 2018–April 2021 (N=632)

*Variable*	*Model 1 AOR (95% CI)*	*Model 2 AOR (95% CI)*	*p*
**Waterpipe use**			
Yes	0.83 (0.59–1.18)	0.85 (0.59–1.22)	0.374
No (ref.)	1	1	
**Group**			
Intervention	1.01 (0.71–1.43)	0.99 (0.69–1.41)	0.941
Control (ref.)	1	1	
**Age** (years)			
<30		1.03 (0.56–1.91)	0.920
30–39 (ref.)		1	
40–49		1.94 (1.16–3.25)	0.011*
50–59		2.26 (1.28–3.97)	0.005*
≥60		4.08 (1.94–8.60)	<0.001*
**Education level**			
Primary school		0.80 (0.39–1.65)	0.547
Completed secondary school (ref.)		1	
Completed high school		0.75 (0.49–1.16)	0.195
Vocational college/University or higher		1.44 (0.85–2.46)	0.179
**Employment**			
Self-employed		1.20 (0.76–1.92)	0.432
Farmer		0.91 (0.56–1.48)	0.693
Other work		0.78 (0.39–1.58)	0.490
Paid work (ref.)		1	
**Comorbidities**			
0 (ref.)		1	
1		0.60 (0.39–0.93)	0.024*
≥2		0.83 (0.53–1.32)	0.430

AOR: adjusted odds ratio. Model 1: controlled for randomization group on smoking cessation outcome, clustering within commune with the outcomes having a binary distribution and using a logit link function. Model 2: as for Model 1 plus age, education level, employment, comorbidities.

* p<0.05.

### WTP and Quitline use among the intervention group only

Among intervention participants, the adjusted models indicated that WTP users had lower odds of verified Quitline use compared to non-WTP users, but this association was not statistically significant (Table 5).

**Table 5:** Association between waterpipe usage and Quitline use among participants in the intervention group using mixed-effect modeling, M2Q2 randomized controlled trial, Vietnam, November 2018–April 2021 (N=369)

*Variable*	*Model 1 OR (95% CI)*	*Model 2 AOR (95% CI)*	*p*
**Waterpipe use**			
Yes	0.65 (0.38–1.11)	0.69 (0.40–1.20)	0.191
No (ref.)	1	1	
**Age (years)**			
<30		0.94 (0.38–2.32)	0.888
30–39 (ref.)		1	
40–49		1.53 (0.71–3.27)	0.274
50–59		0.87 (0.35–2.14)	0.757
≥60		1.04 (0.33–3.27)	0.947
**Education level**			
Primary school		0.62 (0.17–2.26)	0.468
Completed secondary school (ref.)		1	
Completed high school		1.29 (0.67–2.48)	0.443
Vocational college/University or higher		1.54 (0.72–3.32)	0.265
**Employment**			
Self-employed		0.82 (0.39–1.73)	0.602
Farmer		1.00 (0.47–2.11)	0.997
Other work		1.31 (0.46–3.76)	0.610
Paid work (ref.)		1	
**Comorbidities**			
0 (ref.)		1	
1		1.26 (0.63–2.52)	0.506
≥2		1.87 (0.93–3.74)	0.077

Model 1: unadjusted; clustering within commune with the outcomes having a binary distribution and using a logit link function. AOR: adjusted odds ratio. Model 2: controlled for age, education level, employment, comorbidities and randomization group on smoking cessation outcome, clustering within commune with the outcomes having a binary distribution and using a logit link function.

* p<0.05.

## DISCUSSION

Using data from a recently completed smoking cessation trial conducted in rural provinces in Northern Vietnam, we examined who uses WTP and how it may influence smoking behaviors and cessation attempts. We observed age- and education-related differences between WTP users and non-WTP users, with younger adults and those with lower level of education more likely to use waterpipes. Several smoking behaviors also differed between the two groups. WTP users began smoking at a younger age, smoked sooner after waking, spent more on tobacco, and reported lower confidence in their ability to quit. Although WTP use did not affect CO-verified smoking cessation, we observed a non-significant trend suggesting that WTP users were less likely to connect with the Quitline.

Age and education level were inversely related to WTP use, consistent with previous studies identifying the cultural appeal of WTP among younger smokers or its perception as a safer alternative to cigarette smoking, as drivers of use^20-22^. WTP has previously been reported to be associated with higher socio-cultural acceptability, relative inexpensiveness, and perception of reduced harm (compared to cigarette smoking), despite waterpipe tobacco containing significantly higher nicotine content than traditional cigarettes^23,24^. While prior studies have relied primarily on qualitative or small-sample assessments of WTP, our study used high-quality RCT data from 749 individuals who smoke, providing unique insight into WTP use.

WTP users exhibited distinct smoking characteristics compared with non-WTP users, despite broadly similar demographic profiles. Although WTP users smoked fewer cigarettes, they exhibited several proxy indicators such as earlier smoking upon waking, younger age at smoking initiation, higher tobacco expenditures, and lower self-efficacy to quit, suggesting greater potential nicotine dependence. The higher prevalence of tobacco spending among WTP users highlights a greater economic burden, which could serve as a motivator for cessation if leveraged in intervention messaging^6,12^. However, lower self-efficacy among this group may require additional support or tailored engagement strategies to initiate and sustain quit attempts, as self-efficacy appears to improve potential quit attempts^25,26^. A recent meta-analysis explored potential interventions for WTP use, highlighting that counseling and education support may be a good approach to support WTP users^27^. While this appears to be promising, limited research exists on what may be effective for WTP users, as some interventions may utilize strategies used for cigarette cessation, without addressing the unique nuances of WTP use^28^. These behavioral and contextual differences suggest a unique dependence profile that may not be adequately addressed by cigarette-focused cessation programs. Our findings underscore the need for further research to fully understand WTP use and related behaviors in this population, which can provide a stronger framework and context before developing interventions.

Although WTP use was not significantly associated with smoking cessation in our trial, the findings of nicotine dependence by proxy, younger age at smoking initiation, higher tobacco expenditures, and lower self-efficacy to quit, suggest that WTP may impact cessation efforts. Therefore, future studies may be warranted to further evaluate the effect of WTP use on smoking cessation efforts. As previous studies have found that dual use of cigarettes and waterpipes is common^9,11^, especially among younger groups, emphasizing WTP-associated factors may make smoking cessation interventions more effective. Effective strategies may include incorporating WTP-specific feedback in Quitlines, developing messaging that directly challenges misconceptions about WTP’s relative safety, or introducing education and workplace policies that discourage WTP use^27,28^.

Finally, our analysis showed that WTP users had lower odds of using the Quitline, although this association was not statistically significant, potentially due to limited statistical power. Vietnam has made substantial investments to set up these Quitlines, and our findings suggest that WTP users may face additional barriers to engaging with these services. While there are limited studies on WTP users in Vietnam, some data suggest that rates of screening and advice to quit from providers were lower for WTP and dual WTP and cigarette users^29^. Additionally, while Vietnam has invested significant time and effort towards anti-smoking initiatives^30^, little attention has been directed towards WTP use.

### Strengths and limitations

To our knowledge, this is the first study examining sociodemographic associations of WTP use among smokers in rural Northern Vietnam. We report recent RCT data for public health surveillance and policy in Vietnam, where nationally representative data on waterpipe use are limited, especially in rural areas. The study has several limitations. We used data from a parent trial focused on smoking cessation with specific inclusion/exclusion criteria of the study participants and relied on questionnaires, which may limit the generalizability of the findings and introduce potential recall bias. The usage of complete case analysis may have limited our handling of missing cases in our models. Our sample was exclusively male, which, while consistent with the demographics of our target population^16^, may limit overall generalizability. There could have been potential unmeasured factors that we did not collect in the parent study (e.g. strong social norms and ritualistic habits associated with WTP use)^31^ that may impact study findings. Additionally, the potential for reverse causation exists, given that WTP use and smoking-related behaviors were both measured at baseline. Despite these limitations, our findings provide a strong foundation for further understanding behaviors and factors of use among WTP users in Northern Vietnam.

### Public health implications and future directions

Overall, WTP was significantly associated with specific smoking behaviors, such as product usage and smoking frequency, as well as environmental factors like workplace smoking rules, but did not affect smoking cessation outcomes in the context of a mobile health intervention. Given the high prevalence of WTP use among rural smokers in Vietnam, our findings underscore the urgent need for public health campaigns that specifically target dual users, addressing both cigarette and WTP behavior. Future studies may need to focus on qualitative exploration into the perceptions of WTP among smokers, particularly among older persons with established WTP habits, those with lower levels of education, and younger individuals with lower socioeconomic status, in order to develop and test targeted mHealth interventions, such as incorporating specific peer messages designed for waterpipe users. Additionally, studies should focus on establishing a longitudinal relationship to better understand patterns of WTP habits over time. As mHealth smoking cessation interventions expand in LMICs, a nuanced understanding of subgroups like WTP users will be critical to designing effective public health strategies.

## CONCLUSIONS

This study provides insights into specific factors, including current smoking behaviors, which contribute to waterpipe use among smokers in rural Vietnam. Our findings highlight the need for further research into WTP users to better build dependence profiles of WTP use and to develop and test strategies that target WTP and cigarette dual users.

## Data Availability

The data supporting this research are available from the authors on reasonable request.
